# Corona pandemic: awareness of health care providers in Pakistan

**DOI:** 10.3934/publichealth.2020044

**Published:** 2020-07-23

**Authors:** Sadia Minhas, Rabia Mushtaq Chaudhry, Aneequa Sajjad, Iram Manzoor, Atika Masood, Muhammad Kashif

**Affiliations:** 1 Department of Oral Pathology, Akhtar Saeed Medical and Dental College, Lahore, Pakistan; 2 Department of Oral Medicine, Akhtar Saeed Medical and Dental College, Lahore, Pakistan; 3 Department of Community Medicine, Akhtar Saeed Medical and Dental College, Lahore, Pakistan; 4 Department of Histopathology, Akhtar Saeed Medical and Dental College, Lahore, Pakistan; 5 Department of Oral Pathology, Bakhtawar Amin Medical and Dental College, Multan, Pakistan

**Keywords:** COVID-19, health care providers, knowledge, practices

## Abstract

**Introduction:**

Corona pandemic has resulted in a high mortality rate among health care professionals. The purpose of this study was to assess the knowledge and practices of health care providers during this pandemic in Punjab, Pakistan.

**Methods:**

A web based cross sectional survey was conducted during 2^nd^ of April to 20^th^ April 2020 targeting health care professionals working in Punjab, Pakistan. A sample of 540 participants was collected using non probability, convenient sampling technique. Data was generated by using on line google forms after taking IRB approval from institution.

**Results:**

Adequate knowledge was found among health care providers regarding diagnostic tests, modes of transmission, incubation period and preventive strategies. Significant association was seen in knowledge of post graduate and MBBS doctors (Bachelor of Medicine and Bachelor of Surgery) regarding viral etiology (p = 0.006), sign and symptoms (p = 0.000), risk factors (p = 0.000) and mortality rates (p = 0.001). Hand washing was considered as best preventive measure by 95% of the health care providers.

**Conclusion:**

Post graduate doctors have better knowledge regarding corona virus disease transmission, risk factors, incubation period and preventive strategies as compared to undergraduate doctors.

## 1. Introduction

COVID-19 Pandemic was declared by World Health organization on 11^th^ March, 2020 after detection of infection in more than 500,000 cases [1]. There are 38 different species in the Coronaviridae family, latest detection is COVID-19 with highest reported mortality rates [2],[3]. In the past CoV has caused severe acute respiratory syndrome (SARS) in 2002 with 774 deaths globally, and Middle East Respiratory Syndrome (MERS-CoV) causing 858 deaths globally [4]. According to latest report of WHO, 11,500,302 confirmed cases and 535,759 deaths have been reported globally due to this pandemic [5]. COVID-19 outbreak was reported on 12^th^ December, 2019 in Wuhan, China [6]. Pakistan reported the first case of COVID-19 on February 29, 2020, imported the disease through travellers mostly from China and Iran [7].

The incubation period of COVID-19 is estimated to be 4–14 days [8], with most common symptoms like fever, cough and fatigue, along with other less common symptoms headache, sputum, diarrhea, dyspnoea, hemoptysis and lymphopenia [9]. Pneumonia is the most common presentation on a chest CT scan with ground-glass opacities causing death [10]. Hand washing, social distancing, early diagnosis, accurate and timely reporting, quarantine of suspected cases and isolation of confirmed cases are the only preventive measures known till date [11].

In addition to general public, this virus has also targeted health care providers (HCP) due to direct contact and being there as first line of defence in health care emergencies. Although, CDC has outlined guidelines for HCP regarding use of personal protective equipment as well as handling the suspected patients [12].

Still a large number of doctors, nurses and paramedics have suffered from infection and many of them have died during their service to patients [13].

One of the major contributors to decrease mortality rate among HCP during this pandemic can be filling the gaps of existing knowledge. WHO is playing its role in increasing awareness of health care professionals regarding prevention and treatment of this deadly disease. This study aims to assess the awareness levels and practices of HCP regarding COVID-19 in Punjab, Pakistan.

## 2. Methodology

A cross sectional survey was conducted on HCP of Punjab, Pakistan during 2^nd^ of April to 20^th^ April 2020. As it was not possible to do a community-based sampling survey, during this social distancing period, a standardized, electronic questionnaire was used to collect data through web-based survey. Online google surveys (https://docs.google.com) was used through medium of social networking (WhatsApp and Facebook) groups of doctors and students to collect authentic data. Before data collection, the study protocol and procedures were approved by the ethical review committee of Akhtar Saeed Medical & Dental College, Lahore.

A 36-item survey instrument was formulated depending upon the objectives of the study using World Health Organization (WHO) information and detailed search of literature on emerging respiratory viruses (MERS and SARS), as well as COVID-19 data based [14],[15].

Demographic variables included age, gender, marital status, education, employment status and second component was specific to assess knowledge and preventive strategies during this pandemic. A total of 566 participants sent their response. Incomplete forms were discarded (26) and complete forms 540 were included for analysis. The attained data were coded, entered and analysed using SPSS 20 version. Frequencies were presented through tables and graphs. Statistical relationship was assessed among knowledge, attitude and practices of health care professionals in relation to demographic characteristics using Pearson Chi-square test and two-sided Fisher exact test as appropriate. The p value ≤ 0.05 was considered statistically significant.

## 3. Results

Among 540 HCPs, majority were females (n = 382; 70.7%). The most frequent age range was less than 25 years of age (53%). Majority of the study participants were BDS (Bachelor of Dental Surgery) doctors with 23.5%. The detail of other demographic characteristics is given in (Table 1).

**Table 1.** Sociodemographic characteristics of healthcare workers' (N = 540).

Variables	n (%)
Age	
Less than 25 years	286 (53%)
25–34	195 (36.1%)
35–44	39 (7.2%)
45–54	5 (0.9%)
55–64	15 (2.8%)
Gender	
Male	158 (29.3%)
Female	382 (70.7%)
Marital status	
Single	387 (71.7%)
Married	153 (28.3%)
Occupation	
BDS	127 (23.5%)
MBBS	125 (23.1%)
Post-Graduation	110 (20.4%)
BDS student	97 (18%)
MBBS Student	81 (15%)
Workplace	
Hospital settings	470 (87.0%)
Clinic	70 (12.9%)
Source of information	
Television	41 (7.6%)
Social Media	277 (51.3%)
Radio	10 (1.9%)
News Paper	6 (1.1%)
Friends	31 (3.9%)
Television and Social Media	132 (24.4%)
Social Media and Friends	50 (9.3%)
All of the above	3 (0.6%)

Upon inquiring about the main source of information on COVID-19, 51.3% answered social media 277 (51.3%), followed by 10.6% of news media (TV, newspapers, radio) and 21 (3.9%) from friends and family (Table 1).

Most of the participants had general knowledge about aetiology, risk factors, symptoms and transmission of COVID-19, as summarized in (Table 2). The knowledge of HCPs in the present study about COVID-19 was good; 95.9% had heard about COVID-19 and 94.3% considered it infectious whereas, 98.7% knew that causative agent of COVID-19 was a virus. Majority of the participants (99.1%) knew that COVID-19 spreads through droplets and shaking hands with confirmed COVID-19 patient. It was found that participants knew that there was more than one symptom (54.8%) and risk factors (60.7%) associated with COVID-19 infection.

**Table 2.** Knowledge regarding COVID-19 among healthcare workers' (N = 540).

Variables	Number (%)
Heard about COVID-19	
Yes	518 (95.9%)
No	20 (3.7%)
Don't know	2 (0.4%)
Cause of disease	
Virus	533 (98.7%)
Bacteria	5 (0.9%)
Don't know	2 (0.4%)
Novel COVID-19 infectivity	
Yes	509 (94.3%)
No	10 (1.9%)
Don't know	21 (3.9%)
Transmission of disease	
From droplets, touching and shaking hands	535 (99.1%)
Contact with domestic animals	4 (0.7%)
Don't know	1 (0.2%)
Symptoms of COVID-19	
Fever, cough, sore throat	207 (38.3%)
Persistent pain or pressure in chest	15 (2.8%)
Shortness of breath	8 (1.5%)
Pneumonia	7 (1.3%)
All of the above	296 (54.8%)
Don't know	7 (1.3%)
Risk factors associated with COVID-19	
Old age	28 (5.2%)
Diabetes	18 (3.3%)
Respiratory illness	19 (3.5%)
Weak immune	44 (8.1%)
Old age, respiratory illness, weak immune and travel history	63 (11.7%)
Old age, travel history and weak immune	40 (7.4%)
All of the above	328 (60.7%)
Incubation period	
Within 2 days	15 (2.8%)
3–5 days	69 (12.8%)
4–14 days	414 (76.7%)
Don't know	42 (7.8%)
Similarity of COVID-19 with mers and sars	
Yes	136 (25.2%)
No	203 (37.6%)
Don't know	201 (37.2%)
Fatal disease	
Yes	296 (54.8%)
No	222 (41.1%)
Don't know	22 (4.1%)
Diagnostic methods of COVID-19	
PCR	93 (17.2%)
Elisa	18 (3.3%)
Naso-pharyngeal and Oro-pharyngeal swabs	133 (24.6%)
All of the above	185 (34.3%)
Don't know	111 (20.6%)
Mortality rate of COVID-19	
50%	31 (5.7%)
2–3%	336 (62.2%)
10%	69 (12.8%)
Don't know	104 (19.3%)
Vaccine availability for COVID-19	
Yes	67 (12.4%)
No	444 (82.2%)
Don't know	29 (5.4%)
Knowledge of preventive practices of COVID-19 transmission	
Frequent hand washing	36 (6.7%)
Use of hand sanitizers	6 (1.1%)
Wearing face Mask	15 (2.8%)
Frequent hand washing and use of hand sanitizers	104 (19.3%)
All of the above	379 (70.2%)

More than half of the HCPs answered correctly regarding the mortality of the COVID-19 (62.2%) and the period of incubation i.e. 2–14 days (76.7%). On the other hand, only 34.3% of the HCP were aware about the available diagnostic tests of COVID-19. Regarding the knowledge of preventive practices of COVID-19 transmission, a majority of the HCPs (70.2%) agreed that following all measures like handwashing by use of soap, use of hand sanitizers, wearing face mask and observing cough etiquettes are sufficient to avoid COVID-19 infection. About, 82.2% thought that there is no vaccine available but unexpectedly 67 (12.4%) believed in the availability of the COVID-19 vaccine. Around 296 (54.8%) HCP considered COVID-19 a fatal disease with 37.6% answered that COVID-19 is not same as MERS and SARS (Table 2).

**Table 3.** Associations between education level of HCPs and knowledge of COVID-19 by applying two-sided Fisher exact and Pearson Chi-square tests.

Education level						P > 0.05
Items	BDS	MBBS	BDS student	MBBS student	POST-graduation	
Cause of disease						
Virus	98.4%	100%	97.9%	96.3%	100%	p = 0.006
Bacteria	0%	0%	2.1%	3.7%	0%	
Don't know	1.6%	0%	0%	0%	0%	
Heard about COVID-19						
Yes	92.9%	97.6%	93.8%	98.8%	97.3%	p = 0.251
No	5.5%	2.4%	6.2%	1.2%	2.7%	
Don't know	1.6%	0%	0%	0%	0%	
Spread of COVID-19						
From droplets, touching and shaking hands	100%	100%	97.9%	98.8%	98.2%	p = 0.057
Contact with domestic animals	0%	0%	2.1%	0%	1.2%	
Don't know	0%	0%	0%	1.2%	0%	
Signs and symptoms of COVID-19						
Fever, cough and sore throat	36.2%	45.6%	43.3%	27.2%	36.4%	p = 0.000
Persistent pain/pressure in chest	0%	2.4%	0%	2.5%	9.1%	
Shortness of breath	0%	3.4%	0%	3.7%	0.9%	
Pneumonia	0%	0%	0%	3.7%	3.6%	
All of the above	63.8%	46.4%	56.7%	59.3%	49.1%	
Don't know	0%	2.4%	0%	3.7%	0.9%	
Risk factors associated with COVID-19 disease						
Old age	4.7%	6.4%	9.3%	0%	4.5%	p = 0.000
Diabetes	2.4%	7.4%	0%	7.4%	0%	
Respiratory illness	3.1%	8.8%	0%	4.9%	0%	
Weak immune	7.1%	4.8%	13.4%	11.1%	6.4%	
All of the above	63.8%	53.6%	62.9%	60.5%	63.6%	
Old age, respiratory illness, travel history and weak immune	11.8%	15.2%	10.3%	3.7%	14.5%	
Old age, travel history and weak immune	7.1%	4.0%	4.1%	12.3%	10.9%	
Diagnostic test of COVID-19						
PCR	11%	31.2%	11.3%	22.2%	10%	p = 0.000
Elisa	2.4%	2.4%	4.1%	1.2%	6.4%	
Naso-pharyngeal and Oro-pharyngeal swabs	27.6%	16%	27.8%	35.8%	20%	
All of the above	33.1%	30.4%	34.0%	21%	50%	
Don't know	26%	20%	22.7%	19.8%	30.6%	
Mortality of COVID-19						
50%	5.5%	3.2%	8.2%	0%	10.9%	p = 0.001
2–3%	50.4%	69.6%	52.6%	71.6%	69.1%	
10%	16.5%	9.6%	17.5%	13.6%	7.3%	
Don't Know	27.6%	17.6%	21.6%	14.8%	12.7%	

A significant knowledge gap was observed among HCPs (doctors and students) and the knowledge of the study participants differ with education level (Table 3). When two-sided Fisher exact test was applied, a significant association was seen between education level and the knowledge about cause of COVID-19 (p = 0.006). Moreover, a significant association was observed among education level and the knowledge about COVID-19 diagnostic tests (p = 0.000), incubation period (p = 0.001) and mortality (p = 0.002) by applying Pearson chi-square test.

The responses of the participants regarding the various preventive attitudes are given in (Table 4). It was observed that about 70.1% of HCPs had positive preventive attitude towards the prevention of COVID-19 disease. More than 90% of participants (93.9%) practiced frequent hand washing than usual after touching personal items of people with cough, coughing or sneezing, touching door knobs and shaking hands with people, about 73.8% of the participants believed that wearing of surgical mask is not enough for prevention of COVID-19. It means study subjects believe that other preventive practices should also be followed in addition to wearing mask. Actually this finding is in line with higher professional education level of the study subjects.

**Table 4.** Practicing preventive attitudes towards COVID-19.

Items (Agree, Uncertain and Disagree)	Agree	Uncertain	Disagree
Do you think hand washing is necessary for prevention of disease?	507 (93.9%)	29 (5.4%)	4 (0.7%)
Do you think surgical mask can prevent viral transmission?	123 (22.7%)	18 (3.4%)	399 (73.8%)
I am influenced by false news about COVID-19.	294 (54.4%)	71 (13.1%)	175 (32.4%)
Do u think the outbreak of novel COVID-19 is scary	467 (86.5%)	31 (5.7%)	42 (7.8%)
Do you think that COVID-19 infection can be treated at home?	138 (25.6%)	92 (17%)	310 (57.4%)
Being practicing health professionals make you more susceptible to get disease	507 (93.9%)	19 (3.5%)	14 (2.6%)
Do you think that early diagnosis of COVID-19 improve the outcome of the disease?	480 (88.9%)	24 (4.4%)	36 (6.7%)
Do you think health education can prevent COVID-19 infection?	512 (94.8%)	20 (3.7%)	8 (1.4%)

Around 294 (54.4%) of the participants answered that they were influenced by false information about COVID-19 and 86.5% said that the outbreak of COVID-19 is scary. The mainstream of HCPs agreed that COVID-19 patient should not be treated at home (57.4%) with 94.8% believing that health education could help in preventing COVID-19 infections. Overwhelming majority of (88.9%) HCPs think that early diagnosis of COVID-19 can improve the outcome of disease with 93.9% of study participants considered themselves at risk of getting COVID-19 infection.

**Figure 1.**
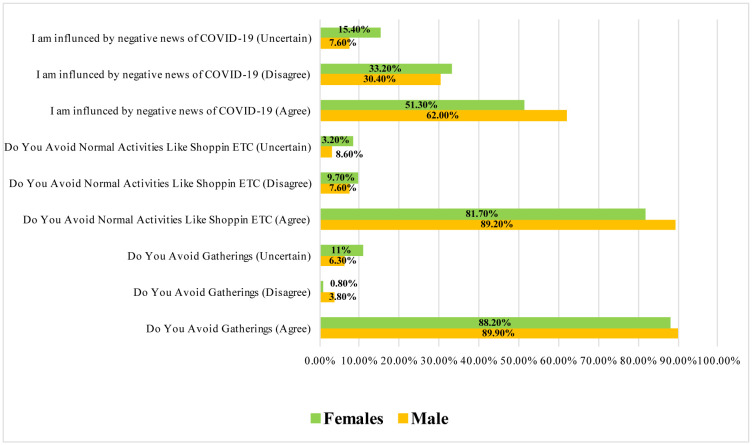
Showing the gender distribution among attitudes towards prevention of COVID-19 (p = 0.013; p = 0.030; p = 0.019).

Items related to COVID-19-related attitude among HCPs in the study were analyzed separately using the Pearson chi-square test to examine their association with gender category (Figure 1).

It was observed that, the male participants showed significantly better attitude towards the possibility of preventing of COVID-19 infection in contrast to females. A significant association was also observed among gender and avoiding gatherings (p = 0.013) and avoid normal activities like shopping, cinemas, travelling etc (p = 0.030) respectively. In general males had better attitude towards COVID-19 preventive measure as compared to females. Regarding influenced by negative news of the COVID-19, the data suggested that males are significantly less influenced as compared to females (p = 0.019).

**Table 5.** Practicing preventive behaviors among healthcare workers' (N = 540).

Items (Yes, Occasionally, No)	Yes	Occasionally	No
Do you avoid gathering?	479 (88.7%)	52 (9.6%)	9 (1.7%)
Are you afraid to go in crowded places?	405 (75%)	57 (10.6%)	78 (14.4%)
Do you avoid normal activities like shopping, cinemas, travelling, school and job?	453 (83.9%)	42 (7.8%)	45 (8.3%)
Do you cover ur nose, eyes and mouth with tissue paper during sneezing and cough?	486 (90%)	23 (4.3%)	31 (5.7%)
Do you throw tissue paper and mask in trash after using it?	522 (96.7%)	18 (3.3%)	0%
Do you eat fruits and vegetables?	137 (25.4%)	382 (70.7%)	21 (3.90%)
Do you use wash ur hands with soap?	520 (96.3%)	20 (3.7%)	0%
Do you use hand sanitizes, hand gels and disposable wipes in absence of water?	248 (45.9%)	240 (44.4%)	52 (9.6%)
Do you wear mask and ensure that It fits properly?	534 (98.8%)	6 (1.1%)	0%
Do you follow the guidelines for treating COVID-19 patients?	513 (95%)	27 (5%)	0%
Are you eager to apply universal infection control precautions?	481 (89%)	59 (11%)	0%
Total	81 %	15.6 %	3.4%

Prevalence of practice parameters among the participants are shown in Table 5. The vast majority of the HCPs were afraid to go in crowded places (75%), avoiding the gatherings (88.7%) and normal activities like shopping, cinemas, travelling, school and jobs (83.9%). The mainstream of participants covers their eyes, nose and mouth with tissue paper (90%) during sneezing and 96.7% of HCPs dispose of the used tissue paper and mask in the trash. The majority of participants (70.7%) occasionally eat vegetable and fruits. In terms of personal protection 98.8% participants reported that they wear mask and after wearing checked their mask weather it fit properly, with majority of participants 520 (96.3%) answered that they washed their hands with soap and about 248 (45.90%) used disinfectants, hand gels and disposable wipes in the absence of water.

The main stream of practicing health professionals (277; 51.3%) refrained themselves from all of these things i.e. being close those who cough or sneeze, shaking hands and touching nose followed by those who cough or sneeze (n = 150; 27.8%), being close and shaking hands 77 (14.3%), shaking hands 17 (3.1%) and touching nose 19 (3.5%). Ninety-five percent of the HCPs answered that they followed the guidelines for treating COVID-19 patients, with eight-nine percent were eager to apply universal infection control precautions.

Items related to practices of COVID-19 among HCPs in the study were analysed by using the Pearson chi-square test to examine their association with gender category. The data suggested that males observed more practices in contrast to females like eating vegetables and fruits (p = 0.017), wearing a mask and ensuring that it fits properly (p = 0.001) and eagerness to apply universal infection control measures (p = 0.013), respectively.

## 4. Discussion

These days COVID-19 is a life-threatening disease spreading throughout the world and has become an international and national issue. COVID-19 pandemic is a major threat to practicing health professionals who are at the forefront of dealing with patients their knowledge, attitude and practices has considerable effects on dealing with COVID-19 patients. This global health emergency, necessitate the study of COVID-19 knowledge, attitude and practice among practicing health professionals [16],[17]. This survey provides an insight on the level of knowledge, attitude, and practices of practicing health professionals on COVID-19 at the time of the outbreak in Pakistan in 2020.

Females were predominant in this survey, which can be described as in Pakistan medical and dental colleges and in hospital settings the number of females health professionals is higher as compared to the male health professional based on the latest data and is similar to the studies conducted in Pakistan and Mumbai [18]–[20]. For the question about sources of information regarding COVID-19, social media (51.3%), was at the highest level, indicating that COVID-19-related updates which are posted online had positive effects on improving level of knowledge of practicing health professionals and as they are more involved in social media rather than official website of Pakistan Ministry of Health, the response is similar to the study conducted in China and Pakistan [18],[21] whereas in contrast to the study conducted in UAE [17]. This is an essential problem for the Pakistan government to transfer knowledge to few of the HCPs who have no knowledge about the COVID-19 outbreak (3.7%) using different information sources.

The current study of 540 of practicing health professionals showed relatively high level of knowledge and positive attitude towards the COVID-19 than previously conducted studies [22],[23], and in accordance to the study conducted in China [21],[24]. We observed that the greatest percentage of participants 95.9% were heard about COVID-19 outbreak and the response is similar to the studies conducted in UAE (97.8%) and China (98.2%) [17],[21]. Regarding the etiological agent majority of participant's knew about it (98.7%) which is similar to the study carried out in China (99.1%) [21].

A positive aspect was observed in the present study that the majority of the participants (99.1%) were answered correctly about the main mode of transmission (droplets and shaking hands) of the COVID-19 which is similar to the surveys carried out in Iran and Jordan (92.9%; 90.5%) [22],[25] and in contrast to the study of Italy (71.4%) [26]. The percentage of practicing health professionals answered about different symptoms were vary considerably with only 54.8% of participants were aware about more than one symptoms of COVID-19, this is in comparison to the studies carried out in Pakistan, Jordan, China, Iran (91.3%; 90%; 72.8%; 90%) [18],[21],[22],[27].

When participants were questioned about features (risk factors) that should be considered to recognize patients at risk of obtaining COVID-19, 60.7% were well aware about the factors associated with the risks of attaining COVID-19 infection which is similar to the studies conducted in China [21],[28]. 76.7% practicing health professionals in this study had good knowledge about the incubation period of COVID-19 (4–14 days) as it is important to know about the exact incubation period because it has part in identifying the safe period for the treatment of suspected patients in hospital and clinical settings and this is similar to the study conducted in Iran (85.4%), whereas in contrast with the study conducted Jordan (36.1%) suggested a knowledge gap among HCPs respectively [22],[25].

Wide-range laboratory tests are available for early diagnosis and successful treatment of suspected COVID-19 patients. Presently, to diagnose the suspected cases real-time reverse transcription-polymerase chain reaction (rRT-PCR) based technique, secretions from lower respiratory tract, oropharyngeal and nasopharyngeal swabs is being used [29],[30]. In the present study, majority of the HCPs were correctly answered about the available diagnostic tests of COVID-19, which is in similar to the study conducted in Iran (80%) [22]. In addition majority of the participant's (82.2%) correctly reported about the unavailability of the COVID-19 which is similar to studies of Jordan (89%) and China (89.3%) [21],[27]. however the study conducted in UAE stated that about 20% of allied health worker believed that flu vaccine can be used for prevention of COVID-19 [17].

About 70.2% of the participants were well aware about the knowledge of prevention of COVID-19 transmission that it can be prevented by maintaining hand hygiene, wearing face mask and cough etiquette's which is in concordant to the study conducted in UAE (85.6%), Iran (93.8%) and China (98.2%) [17],[21],[22], with 54.8% of practicing health professionals thought that COVID-19 is fatal which is in contrast to the study conducted in China (98.8%) and UAE(88.5%) [17],[21].

A significant association was observed between education level and awareness regarding the available diagnostic test of COVID-19 (p = 0.000), incubation period of COVID-19 (p = 0.001) and mortality of COVID-19 (p = 0.002), highlighting the fact that education has positive impact on person's knowledge about COVID-19 and these findings are in accordance with the studies conducted in UAE [17].

Another significant result of the present study was that the considerable number of practicing health professionals 70.1% had a positive and different attitude about COVID-19. During the pandemic of COVID-19, it has been continuously highlighted the significance of hand washing and in the case of practicing health professional this plays a crucial part in the prevention of spread of disease. The majority of the 93.9% of participant's reported that washing hands frequently can aid in the prevention of transmission COVID-19 from suspected or identified patients, this is similar with the survey's conducted in Iran (96.7%) and Jordan (99.7; 96.2%) [22],[25],[27] and almost 73.8% reported that wearing of surgical masks is not enough to reduce the chances of transmission of COVID-19 infection to themselves and patients were in contrast with the Jordan and Iran study (85.5%; 68.4%) [22],[27].

About 54.4% of the HCPs were influenced by false news, WHO recommended to decrease that listening and watching of news about COVID-19 as it causes worriness and anxiety so, only depend on reliable sources [29] with 86.5% of participants were scared about outbreak of COVID-19 and this is strengthened by the study conducted in Italy revealing that 52% of HCPs were quite worried about COVID-19 [26].

The current study revealed that maximum number of participants (93.9%) were afraid of getting themselves infected with COVID-19 infection with maximum numbers of the practicing health professionals are afraid of treating any patient with suspected symptoms of COVID-19, this response is similar to the perception of other studies conducted in China 82.3% and 85% where HCPs were afraid of getting infected at work [21],[32]. The majority 512 (94.8%) reported that it is significant to give health education to people about COVID-19 infection to prevent the spread of the disease, similar to the study carried in Jordan (97.8%) [25].

Generally, most HCP had a positive attitude towards prevention and control of COVID-19. However, differences were identified in the attitudes among gender, in which males showed significantly better attitude towards COVID-19 prevention (p = 0.013; p = 0.030) and this is in accordance to the study conducted in China (p = 0.019) [33].

In order to evaluate the practices surveyed by the participants to prevent COVID-19 infection, a vast majority of practicing health professional good practices towards the COVID-19 preventive measures. The majority of the practicing health professionals were afraid to go in crowded places (75%) this is comparable to the studies of Iran (99.6%), Italy (41.4%) and China (96.4%) [22],[26],[33]. In present study the participants avoided gatherings (88.7%) and normal activities (83.9%) and is comparable with the study of Iran (99.6%; 95%; 94.6%) [22]. This preventive practices towards COVID-19 was due to raised knowledge among HCP because of increased infectivity and transmission of the COVID-19 and these strict preventive measures or education are implemented by government, while 14.5% of HCP disagreed with this preventive practice towards COVID-19.

The attitude participants on practices reveals the particular path to avoid the COVID-19 by multiple means like eating vegetables and fruits (25.4%), washing hands with soap (96.3%), wearing mask (98.8%), avoiding hand shaking and following preventive sneezing and cough manners (51.3%) are well recognised in the prevention of respiratory infectious diseases, these findings are similar to other multiple relevant studies suggesting the fact that health care professionals has higher level of awareness of the actions recommended and following the suggested practices [17],[21],[22],[26],[27],[34].

The mainstream of participants covers their eyes, nose and mouth with tissue paper (90%) during sneezing and 96.7% of HCPs dispose of the used tissue paper and mask in the trash and in accordance with the study conducted by Alzoubi and his colleagues (95.8%) [27].

Ninety-five of participants followed the guidelines (standards, contact and airborne precautions) proposed by national and international authorities for treating confirmed and suspected COVID-19 patients in hospital and clinical settings, as it is important in this time of pandemic to follow the guidelines and minimize the chances of spread and is similar to the studies carried out India (79%) and Jordan (90%), and in contrast to the Italian (41%) study [19],[25],[26]. Similarly, it was motivating that more than 80% of participants were aware and eager to apply infection control measures (cough etiquettes, hand hygeine, well ventilated and separate waiting area, respiratory hygiene) for suspected COVID-19 patients, which is similar to the study conducted in India (75%) [19].

To best of our knowledge, this is the first study which assesses the knowledge of COVID-19 among Punjab, practicing health professionals. The attitude and practice of practicing health professional about getting infected from COVID-19 could be significantly decreased if health professionals precisely follow the applicable recommendation issued by the authorities, along with additional precautions if patients presented with any suspicious signs and symptoms.

We recognise that our study has few limitations, which includes the fact that the questionnaire was administered online with limited duration of time and have not been validated before due to novelty of COVID-19. Another limitation was difficulty in interpretation of results as there is very limited research to compare with on knowledge, attitude and practice of HCPs on COVID-19.

## 5. Conclusion

To conclude, the present study revealed a high level of knowledge, positive perception and attitude and satisfactory preventive practices among practicing health professionals. Male have better attitude and practices towards COVID-19. The results reflect the effect of COVID-19 declaration as pandemic announced by WHO and local health authorities to follow the guide lines and infection control measure in avoiding spreading of COVID-19. This study data can also aid the health policy maker of Pakistan in the improved planning and devising of the health policies as well as to focus on effective risk communication and education for the control of epidemics, especially for vulnerable groups like HCPs. This knowledge, attitude and practice may be one of the reasons of less than expected spread of COVID-19 in Pakistan, so similarly researches in other settings and larger population need to be conducted.
