# Supplementary material for: Corona pandemic: awareness of health care providers in Pakistan
Source: AIMS Public Health. 2020 Jul 23;7(3):548–61. doi: 10.3934/publichealth.2020044 (PMC7505782; doi:10.3934/publichealth.2020044)
Supplement: Supplementary file 1 [file publichealth-07-03-044-s001.pdf]

Public Health

AIMS Public Health, 7(3):xx–xx.
DOI: 10.3934/publichealth.20200xx
Received: 10 May 2020
Accepted: 20 July 2020
Published: xx 2020

http://www.aimspress.com/journal/aimsph

Research article

Corona pandemic: awareness of health care providers in Pakistan

Sadia Minhas[1], Rabia Mushtaq Chaudhry[1], Aneequa Sajjad[1], Iram Manzoor[2], Atika Masood[1] and Muhammad Kashif[3,*]

[1] Department of Oral Pathology, Akhtar Saeed Medical and Dental College, Lahore, Pakistan
[2] Department of Community Medicine, Akhtar Saeed Medical and Dental College, Lahore, Pakistan
[3] Department of Oral Pathology, Bakhtawar Amin Medical and Dental College, Multan, Pakistan

* **Correspondence:** Email: drkashifazam@gmail.com; Tel: +00923346035054.

Supplementary

The attitudes and degree of aw areness about novel-COV19 among health care w orkers of punjab (doctors, nurses and technicians) demographic:

Age:
15–24 25–34 35–44 45–54 55–64

Gender:
Male Female

Marital status
Married Single Divorced Widowed

Educational level
MBBS student BDS student MBBS BDS Post Graduation

Workplace
Hospital setting Clinical settings

The main source of information u get from:
Television Social Media Radio Newspaper Friends All of the Above

Knowledge about the novel COVID-19:

Have you heard about novel COVID-19?
Yes No Don't Know

The cause of disease is:
Virus Inherited Bacteria Don't know

Is novel COVID-19 infectious?
Yes No Don't Know

The transmission of disease is:
From droplets and touching and shaking hands Sexual route Fast food
Contact with domestic animals Don't Know

What are symptoms of disease?
Fever, cough, sore throat Persistent Pain or pressure in the chest
Shortness of breath Pneumonia All of above Don't know

How long does it take to symptoms of the disease to appear after infection?
Within 2 days from 3 to 5 days 4–14 days Don't know

The risk factors for novel covid 19 includes:
Old age Diabetes Respiratory Illness
Weak Immune Pregnant Females Recent Travel History
All of the above Don't Know

Novel COVID-19 is a fatal disease to any individual?
Yes No Don't know

Is novel COVID-19 is same as mers and sars?
Yes No Don't know

Diagnostic method of novel COVID-19 includes:
ELISA PCR Nasopharyngeal Oro-pharyngeal /swabs
All of the above None of the above

The following practices can avoid novel COVID-19 transmission (change question in survey)
Frequent Hand Washing Use of Hand Sanitizers
Covering your nose with ur hands during coughing and sneezing
Wearing Face Mask All of the Above

Do you know the mortality rate of the disease?
50% 2–3% 10% don't know

Is there a vaccine for novel COVID-19?
Yes No Don't know

Attitude towards the prevention of novel COVID-19

Do you think hand washing is necessary for prevention of disease?
Agree Uncertain Disagree

Do you think that COVID-19 infection can be treated at home?
Agree Uncertain Disagree

Being practicing health professionals make you more susceptible to get disease?
Agree Uncertain Disagree

DO you think that early diagnosis improves the outcome of disease?
Agree Uncertain Disagree

Do you think that health education can prevent the novel COVID-19?
Agree Uncertain Disagree

I am influenced by false news about novel COVID-19
Agree Uncertain Disagree

DO you think outbreak of novel COVID-19 is scary?
Agree Uncertain Disagree

Practice about novel COVID-19

Do you avoid gatherings?
Yes Occasionally No

Are you afraid to go in crowded places?
Yes Occasionally No

Do you avoid normal activities like shopping, cinemas, travelling, school and job?
Yes Occasionally No

Do you cover ur nose, eyes and mouth with tissue paper during sneezing and cough?
Yes Occasionally No

Do you throw tissue paper and mask in trash after using it?
Yes Occasionally No

I do you eat fruits and vegetables
Yes Occasionally No

Do you wash ur hands with soap?
Yes Occasionally No

Do you use hand sanitizes, hand gels and disposable wipes in absence of water?
Yes Occasionally No

Do you wear mask and ensure that it fits properly?

Yes Occasionally No

Do you follow the guidelines for treating COVID-19 patients?

Yes Occasionally No

Are you eager to apply universal infection control precautions?

Yes Occasionally No
